# Supplementary material for: The Incorporation of Graphene Nanoplatelets in Tung Oil–Urea Formaldehyde Microcapsules: A Paradigm Shift in Physicochemical Enhancement
Source: Polymers (Basel). 2024 Mar 26;16(7):909. doi: 10.3390/polym16070909 (PMC11013791; doi:10.3390/polym16070909)
Supplement: Supplementary file 1 [file polymers-16-00909-s001.zip › polymers-2730683-supplementary.pdf]

Supplementary Data

The Incorporation of Graphene Nanoplatelets in Tung Oil–Urea Formaldehyde Microcapsules: A Paradigm Shift in Physicochemical Enhancement

Abdullah Naseer Mustapha *, Maitha Almheiri, Nujood AlShehhi, Nitul Rajput, Zineb Matouk and Nataša Tomić

Advanced Materials Research Centre (AMRC), Technology Innovation Institute (TII), Masdar City, Abu Dhabi, United Arab Emirates
* Correspondence: author: abdullahi.mustapha@tii.ae

1. Experimental methods

1.1. X-ray photoelectron spectroscopy (XPS)

X-ray photoelectron spectroscopy (XPS) is a quantitative technique that uses the photoelectric effect to identify elements within a material, their overall electronic structure, and their chemical state. XPS was performed using a PHI VersaProbe 5000 Scanning X-ray Photoelectron Spectrometer. A monochromated Al X-ray source (1486.6 eV) was used as a probe for the experiments. The X-ray beam power was 50.17 W with a step size of 0.05 eV, and detector pass energy of 280 eV. During the experiment, E-neutralizer (1V) and I-neutralizer (0.11 kV Ar+ ion) were implemented. The compositions were calculated by using the area under the high resolution and weighted with the respective sensitivity factors for each elemental species. Peaks were calibrated using the C1s as reference at 284.6 eV. The software Casa XPS was used for curve fitting and calibration. The GNP/TO samples were prepared using a Hielscher UP400St ultrasonic homogenizer at 30% amplitude for 5 min and then the mixture was repeatedly centrifuged and washed with dimethylformamide (DMF). After the washing process, the particles were dried at 80 °C under a vacuum for 48 h. This way, all unreacted TO is removed from the surface of the GNP particles, so it can be seen more clearly if the TO during the ultrasonic homogenization process affected the structure of the GNPs.

1.2. (X-ray diffraction) XRD

Powder XRD analysis was used to investigate the interaction of GNPs and TO that can occur during the synthesis of MCs. XRD can be used to compare the crystallinity of substances to see the effect of additives on the structural changes. The tests were recorded on a Bruker D8 Advance instrument (Bruker, Germany). Cu-Kα radiations were employed as an X-ray source (λ = 0.154 nm). The test was run in the 0-60° range at room temperature, with a 0.01° increment and 0.5 s/step. The GNP/TO samples were prepared the same way as the ones for XPS.

1.3. Contact Angle

To study the contact angle for the TO and GNP/TO droplets, a Rame-Hart Automated Goniometer (model no. 590-u4), with 20 μL drops of each sample was used in each measurement.

1.4. Gel Permeation Chromatography (GPC)

Viscotek TDA 305 and GPC max from Malvern Panalytical, UK, were used to analyze the influence of GNPs on the molecular weight of the PU shell of obtained MCs. The detector used in the analysis was Refractive Index (RI) with TSK gel GMPWXL x 2 SEC/GPC Columns at 1.0 mL.min^{-1} flow rate at 30 °C. The calibration standard was PS at 1.0 - 3.0 mg.mL^{-1} concentration.

To prepare the samples for GPC analysis, 2 mL of each solution was freeze-dried at room temperature overnight (at least 18 hours) to remove the solvent and isolate the polymer and other non-volatile components. The residues were weighed and dissolved in 2 mL of THF, then left on the rocker at room temperature overnight (at least 18 hours) to dissolve. Then, they were diluted to a 30 mg/mL concentration in THF. The samples were filtered through 0.22 μm PTFE syringe filters and injected for GPC analysis. The analysis was performed in 3 injections per sample.

All individual injection GPC results were processed by setting baselines and limits for the polymer peak using the OmniSEC Software Version 4.6.2.

2. Results

2.1. XPS analysis

The surface compositions of the pristine GNPs and functionalized GNP-TO were analyzed by XPS. Figure S1 shows the high-resolution C 1s spectra of the pristine (a) and GNP-TO (b). The XPS spectrum of the pristine GNP peaks corresponds to the binding energies of about 284 eV and 531 eV, which are attributed to C1s and O 1s energy levels, respectively. The oxygen content in the pristine GNPs was found to be around (7%) with respect to carbon. The contributions at 284.5 eV (major contribution, red) and smaller contributions at 285 eV (green), 286.1 eV (yellow), and 290.2 eV (magenta) arising from the C=C (sp2 bonded carbons), C-C (sp3 bonded carbons), and C-OH (alkoxy) are characteristics of the C1s core-level spectrum of GNPs (a). The weakly pronounced peak (violet curve) of the C1s shape suggests a shape close to pure GNPs, compatible with the information described above. The shape of the C1s indicates a similar shape to pure GNPs, which is consistent with the above-mentioned information. The small oxygen content can also be attributed to surface contamination [1].

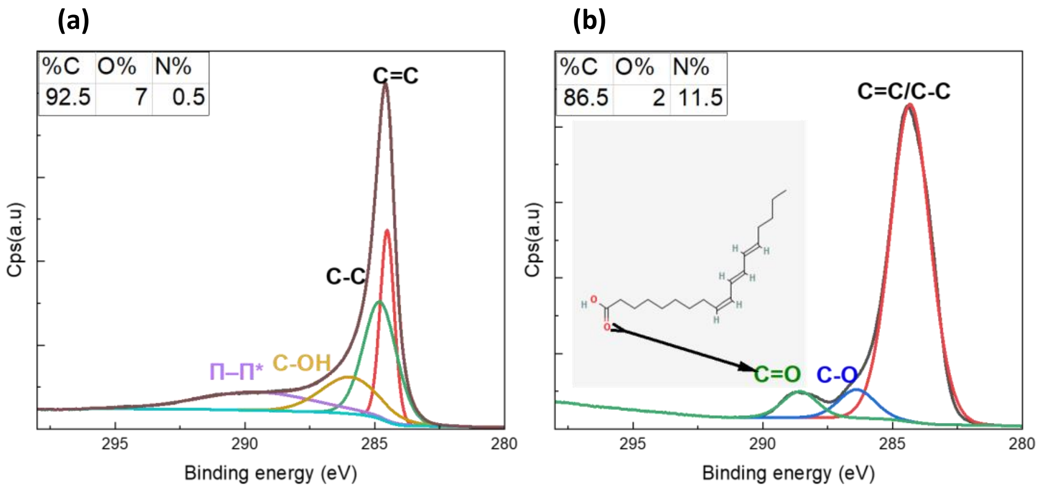

Figure S1. The C 1s core-level spectrum of (a) GNP, (b) GNP/TO containing chemical composition determined by XPS.

The functionalized GNP/TO XPS spectra exhibit peaks with binding energies of around 284 eV, 399 eV, and 531 eV, respectively, which are attributed to the C1s, N 1s, and O 1s energy levels. When compared to the pristine GNP material, the functionalized samples also exhibit a notable increase in nitrogen (up to 11.5%). This can be attributed to the traces of DMF solvent used to remove unreacted TO. All the atomic concentration percentages for the various

chemical elements are depicted in Figure S1 (b). When compared to the pristine GNPs, the GNP-TO spectra become more asymmetric, which is connected to the rise in contributions at 284.5 eV (red) and 286.1 eV (blue), as previously indicated. At 288.9 eV (green curve), a novel contribution originating from the O-C=O (carboxyl) moieties formed from the carboxylic acid functionality is readily discernible. The XPS results suggested that during the homogenization process of GNPs and TO, the chemical reaction between GNPs and TO occurred, either through esterification reactions or double bond cross-linking. These results indicate good compatibility of the mentioned components and good dispersion of GNPs in TO.

2.2. XRD analysis

Figure illustrates the findings of a study conducted to determine the crystallinity of the pristine GNPs and the TO-modified GNPs (GNP/TO) using XRD. According to Figure S2, the XRD pattern demonstrates crystalline character, with a powerful diffraction peak at around 26.6°. It is typical for the distinctive peak of the graphitic compound in the form of graphene multilayers, which can be seen in the XRD pattern of both samples. A reduced peak intensity, in the case of GNP/TO, is observed. All diffractions corresponding to GNPs were still visible for the GNP/TO although their intensity decreased and lost some of their sharpness. This displays the modified TO reacting with the surface of the GNPs to form GNP/TO.

Additionally, the GNP/TO conveyed a homogeneous layer of TO matrix covering them since the modified GNP/TO characteristic peak was wider than the unmodified GNPs [2]. The d-spacing can then be calculated from Bragg's law:

Reflection (n) × Wavelength (λ) = 2 × Interplanar spacing (d) × Sinθ

So, interplanar spacing or d-spacing for both samples could be calculated as

Interplanar spacing (d) = Order of Reflection (n) × Wavelength (λ) / 2 × Sinθ

In our case, this can be calculated by taking reflection order = 1 and wavelength λ = 0.154, so the d-spacing should be d = 0.34 nm for both GNPs and GNP/TO. These results indicated that after the ultrasonic homogenization of GNPs and TO, no structural changes, such as

interplanar spacing, happened in the GNPs. Nevertheless, the results from XRD were in accordance with the XPS results, confirming the interaction between GNPs and TO.

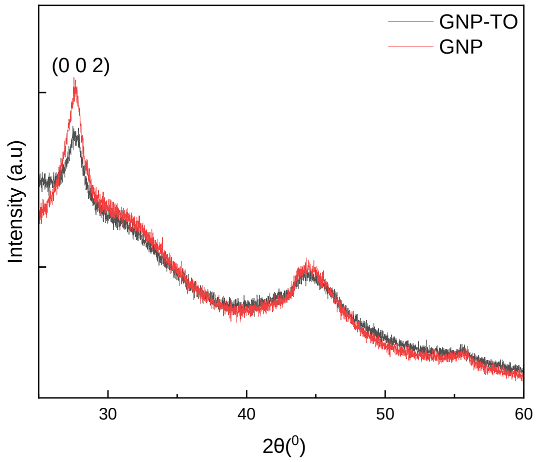

Figure S2. The XRD patterns of the pristine GNPs (red line) and the modified GNP-TO (black line).

2.3. Contact Angle

While the encapsulation process is immediate, the measurement of the contact angle over a period of four days provides critical insights into the post-encapsulation behaviour of microcapsules, particularly in terms of adhesion and stability on different substrates. In this study, the content of MCs, i.e., the mixture of TO and GNPs, was investigated. The reason for this is that when the damage happens in the coating, the core content is released and spreads over the metal surface exposed due to the damage (see Figure S3). Higher contact angles generally cause poor wetting of substrates and the appearance of air gaps that can present the target locations for corrosion. Thus, the contact angle measurements of TO with different percentages of GNP were performed on a glass slide over a period of 4 days, as seen in Figure S4. Figure S4 (a) shows that at zero time, the value of the contact angle increased with the addition of GNP. But, after 1 day, the trend reversed. So, the sample with 5 wt. % GNP had the lowest contact angle value, while the pure TO and TO + 1 wt. % GNP had the same values. On the 4th day, the differences were more emphasized, showing that pure TO exhibited the highest contact angle, while higher percentages of GNPs resulted in lower contact angle measurements. Figure S4 (b) represents the contact angle values dependent on the amount of

GNPs used. The linear fit exhibited a perfect correlation with $R^2 = 1$. The reason for the reduction in the contact angle in the first stage (1 day) can be associated with improved wetting properties of TO over time. For samples with GNPs, there was another phenomenon associated with the precipitation of GNPs in the contact surface with the glass slide. This way, the adhesion with the glass was improved, and thus, the contact angle was therefore reduced. As time elapsed (4 days), this enabled enough time to achieve appropriate precipitation and a clear difference between the samples. The results obtained from the contact angle measurements suggested that the presence of graphene can improve both the adhesion and the corrosion protection of coatings with GNP/TO MCs.

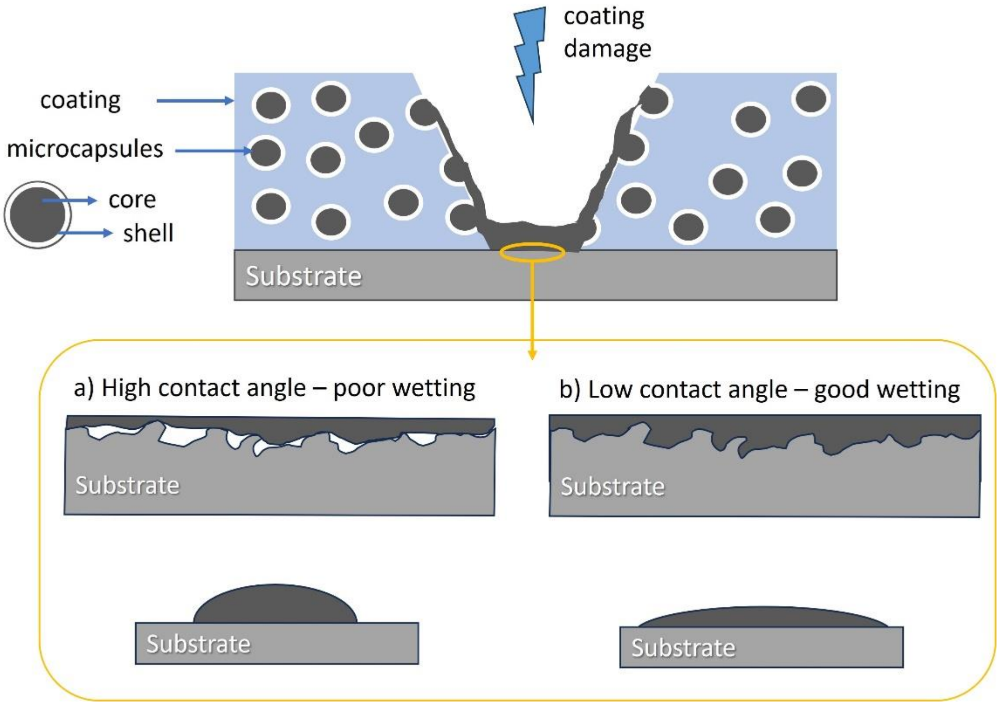

Figure S3. Schematic representation of the influence of contact angle values on the wetting of MCs content, i.e., GNP/TO.

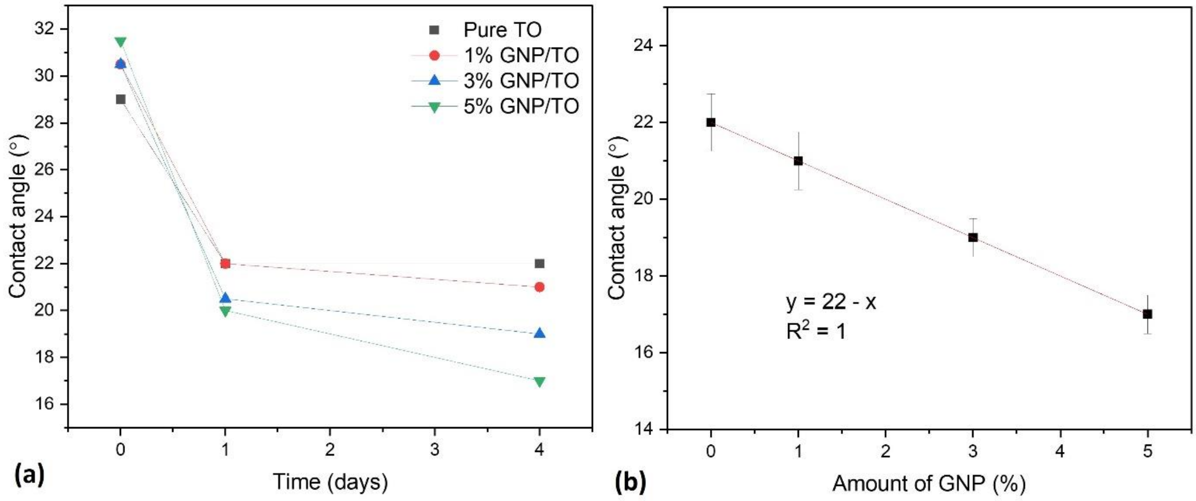

Figure S4. The contact angle of TO droplets with different amounts of GNPs on a glass slide: (a) monitored for a duration of 4 days, and (b) the linear correlation between the contact angle and the amount of GNPs.

2.4. GPC analysis

During GPC analysis, two peaks were observed in all the sample chromatograms (see Figure S5). The first peak (at lower retention time, i.e., higher molecular weight) has a M_w of 16179 Da in the pure sample, 35992 Da in the 1% GNP sample, 34196 Da in the 3% GNP sample, and 24929 Da in the 5% GNP sample. The second, much larger peak has a M_w of approx. 1300 Da in each sample and an extremely narrow polydispersity, suggesting it belongs to a small-molecule (non-polymeric) component of the sample mixtures.

It is noted that the M_w increases with GNP content at 1 and 3% vs. the control sample, which can arise from aggregates present in the sample. For this, overlays of GPC-RI and GPC-LS from the first injection samples are plotted to identify the presence of aggregation (see Figure S5). Comparing the GPC-RI chromatograms with the corresponding GPC-LS chromatograms confirmed the presence of aggregates at 12 min retention time. Interestingly, the intensity of the GPC-LS peak is higher in GNP 1 % and 3 % compared to pure and 5 % GNP samples, which explains the higher M_w values in samples containing 1 and 3 % GNP.

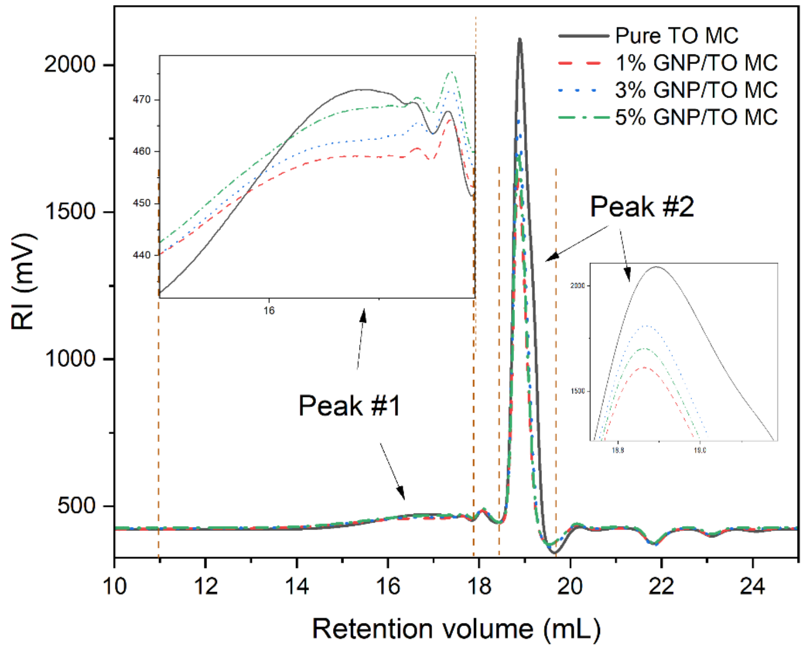

Figure S5. Comparative GPC-RI chromatogram for all the samples with the two observed peaks in the signal.

Table S1. GPC results showing M_n, M_w, and PDI for the two observed peaks.

Sample	Peak #	M_n (Da)	RSD (%)	M_w (Da)	RSD (%)	M_w/M_n (PDI)	RSD (%)
Pure TO MC	1	9278	0.02	16179	4.06	1.744	4.51
	2	1250	0.46	1280	0.45	1.025	0.05
1% GNP/TO MC	1	10737	0.77	35992	6.93	3.354	7.65
	2	1333	0.64	1355	0.66	1.016	0
3% GNP/TO MC	1	10397	2.15	34196	2.88	3.292	4.68
	2	1320	0.19	1341	0.15	1.017	0.08
5% GNP/TO MC	1	9966	0.73	24929	6.98	2.502	7.46
	2	1323	0.34	1344	0.33	1.017	0.05

References:

1. Cunha, E.; Ren, H.; Lin, F.; Kinloch, I.A.; Sun, Q.; Fan, Z.; Young, R.J. The Chemical Functionalization of Graphene Nanoplatelets through Solvent-Free Reaction. RSC Adv. 2018, 8, 33564–33573, doi:10.1039/C8RA04817G.

2. Anand, G.; Odillard, L.; Kibet, A.M. Performance Optimization by Study of Tribological and Rheological Properties of Dates Oil with Graphene Oxide Nano-Additives. https://doi.org/10.1080/17597269.2022.2079248 2022, 1–7, doi:10.1080/17597269.2022.2079248.
